# A Genetic Population Isolate in The Netherlands Showing Extensive Haplotype Sharing and Long Regions of Homozygosity

**DOI:** 10.3390/genes8050133

**Published:** 2017-05-04

**Authors:** Metten Somers, Loes M. Olde Loohuis, Maartje F. Aukes, Bogdan Pasaniuc, Kees C. L. de Visser, René S. Kahn, Iris E. Sommer, Roel A. Ophoff

**Affiliations:** 1 Brain Center Rudolf Magnus, Department of Psychiatry, University Medical Center Utrecht, Utrecht 3584 CX, The Netherlands; M.Somers@umcutrecht.nl (M.S.); maartje.aukes@knaw.nl (M.F.A.); r.kahn@umcutrecht.nl (R.S.K.); i.sommer@umcutrecht.nl (I.E.S.); 2 Center for Neurobehavioral Genetics, Semel Institute for Neuroscience and Human Behavior, University of California Los Angeles, Los Angeles, CA 90095, USA; loldeloohuis@mednet.ucla.edu; 3 Department of Human Genetics, David Geffen School of Medicine at UCLA, University of California Los Angeles, Los Angeles, CA 90095, USA; pasaniuc@ucla.edu; 4 Department of Pathology and Laboratory Medicine, University of California Los Angeles, Los Angeles, CA 90095, USA; 5 Department of General Practice, University Medical Center Groningen, University of Groningen, Groningen 9713 GZ, The Netherland; KdVisser@hetdokurk.nl

**Keywords:** genetic isolate, linkage disequilibrium, runs of homozygosity, IBD sharing, effective population size

## Abstract

Genetic isolated populations have features that may facilitate genetic analyses and can be leveraged to improve power of mapping genes to complex traits. Our aim was to test the extent to which a population with a former history of geographic isolation and religious endogamy, and currently with one of the highest fertility rates in The Netherlands, shows signs of genetic isolation. For this purpose, genome-wide genotype data was collected of 72 unrelated individuals from this population as well as in a sample of 104 random control subjects from The Netherlands. Additional reference data from different populations and population isolates was available through HapMap and the Human Genome Diversity Project. We performed a number of analyses to compare the genetic structure between these populations: we calculated the pairwise genetic distance between populations, examined the extent of identical-by-descent (IBD) sharing and estimated the effective population size. Genetic analysis of this population showed consistent patterns of a population isolate at all levels tested. We confirmed that this population is most closely related to the Dutch control subjects, and detected high levels of IBD sharing and runs of homozygosity at equal or even higher levels than observed in previously described population isolates. The effective population size of this population was estimated to be several orders of magnitude smaller than that of the Dutch control sample. We conclude that the geographic isolation of this population combined with rapid population growth has resulted in a genetic isolate with great potential value for future genetic studies.

## 1. Introduction

Population isolates have become increasingly valuable for genetic research [1,2,3,4]. As a result of founder events followed by subsequent rapid expansion, these populations manifest reduced genetic diversity, characterized by differences in allele frequency patterns and haplotype distribution, increased identical-by-descent (IBD) sharing within the population as well as extended chromosomal regions of homozygosity (ROHs) [1,2,5,6,7,8,9]. Population isolates tend to show less variation in lifestyle and other environmental factors, which also reduces non-genetic variance [1]. Because of these properties, genetically isolated populations are likely to facilitate gene mapping of human traits, and can be leveraged to improve power of genome wide association studies. In particular, rare variants may have drifted up in frequency thereby improving power to establish low-frequency and rare variant associations with complex phenotypes [10,11,12,13,14,15,16].

Genetic isolation is frequently caused by geographic boundaries, such as has been described for populations from the Central Valley of Costa Rica [17], the Val Borbera valley in Italy’s Piedmont region [18] and Sardinia [19]. Genetic isolation as a result of social and religious endogamy rather than geographical barriers, has also been shown within The Netherlands [5,20]. The advantages of such a well-characterized and genetically isolated population became apparent in a genetic mapping study of a rare recessive Mendelian liver disorder [20] as well as for the complex trait of human height [3]. Further exploration of regional population histories in The Netherlands as well as other parts of Europe is likely to yield additional population isolates with advantages for genetic studies of human traits.

We recently performed a genetic linkage study of left-handedness and language lateralization in a town with one of the highest fertility rates currently in The Netherlands, which is an advantage for collecting large kindreds [21]. This town (which we abbreviate to TWN), was an inhabited island for many centuries until it became connected to the mainland in the first half of the 20th century. For this reason, we hypothesized that the TWN population might represent a genetic isolate. We investigated the genetic features of this population and examined whether it shows signs of genetic isolation compared to a random sample from The Netherlands, HapMap [22] and other population isolates represented in the Human Genome Diversity Project (HGDP) [23,24]. Genetic analyses of TWN showed significant features of a population isolate that is most closely related to a control sample from The Netherlands, but with extensive measures of haplotype sharing, increased rates of ROHs, and a very small effective population size similar to previously reported genetic isolates.

## 2. Materials and Methods

### 2.1. Populations and Genotype Data

Historical records indicate that the TWN population is relatively young: the island was first inhabited around 1000 A.D. and its population was reduced to 151 inhabitants after a bottleneck event in the seventeenth century due to an infectious disease. The current population extends to 19,500 individuals. The demography of the town is still characterized by strong social and religious coherence. The fertility rate is high (>3.0 children per mother) compared to average levels in The Netherlands (1.68) (Dutch Bureau for Statistics). A total of 72 founders were selected from 37 multi-generational families that were ascertained on the basis of high prevalence of left-handedness and participated in a study on language lateralization and left-handedness [21]. A set of 104 Dutch controls (NLD) that were previously recruited for genetic studies of neuropsychiatric traits [25] was used as reference population. Both datasets were genotyped using the 330k_CytoSNP array (Illumina Inc., San Diego, CA, USA), resulting in genome-wide genotype data of almost 300,000 Single Nucleotide Polymorphims (SNPs). The Medical Ethical Committee of the UMCU (Utrecht, The Netherlands) approved of the mentioned studies (identification code 06/083, approval date June 27, 2006) and written informed consent was obtained from all participants according to the declaration of Helsinki.

A number of reference datasets were included for comparison: from the HapMap III (HM3) [22] dataset, we included Utah residents with Northern and Western European ancestry from the CEPH (formerly, the Centre d'Etude du Polymorphisme Humain) collection (CEU) and Tuscans in Italy (TSI) populations; for HM3 datasets 1,025,841 genotyped SNPs were available. Further, European reference populations with known features of genetic isolation were included from the HGDP [23,24]: the Sardinian (*n* = 28), French (*n* = 27), Russian (*n* = 25), Basque (*n* = 24), Adygei (*n* = 17), Orcadian, (*n* = 14), Italy-Bergamo (*n* = 12), and Tuscan (*n* = 8) populations; for the HGDP samples we had access to 660,918 genome-wide genotyped SNPs.

### 2.2. Data Analysis

Randomly selected subjects within an isolated population are more likely to be closely related. Since relatedness of individuals may interfere with our outcome measures of isolation (e.g., IBD sharing), we removed closely related individuals and performed stringent quality control as described in Appendix A. In particular, while all individuals from TWN were self-reported unrelated, we removed 13 samples related up to the level of third degree relatives (with PI-hat > 0.1), compared to 1 sample from NLD that was related to another NLD sample. After quality control, we included the following sample sizes in our analysis: 59 TWN, 96 NLD, 102 EU, 81 TSI, 25 French, 23 Basque, 28 Sardinian, 25 Russian, 16 Adygei, 12 Italy-Bergamo and 12 Orcadian samples, as well as a total of 112,444 SNPS. For analyses involving only NLD and TWN, these numbers are 58 individuals from TWN, 92 from the NLD, and 254,582 SNPs.

### 2.3. Measure of Population Stratification

We performed multidimensional scaling (MDS) analysis using the PLINK module by extracting the first four dimensions based on the IBS matrix [26]. As MDS analysis assumes markers to be independent we used the SNP dataset that remained after pruning (53,490 independent SNPs for the 12 merged datasets with r^2^ < 0.2).

### 2.4. Pairwise Genetic Distance

The F_ST_, indicating genetic differentiation expressed by variation in allele frequency [27], was calculated for each pair of populations. The F_ST_ was calculated using the merged population datasets (112,444 SNPs) using the R package HIERFSTAT [28]. Because of computational constraints 100 permutations were performed to determine *p*-values and confidence intervals.

### 2.5. Decay of Linkage Disequilibrium (LD)

Decay of LD by genomic distance was estimated between markers. To control for sample size, we performed the analyses using sets of approximately equal sample sizes by randomly selecting 25 subjects from each population, leaving the smaller HGDP sets out of the analysis. Pairwise LD was calculated with PLINK using the squared correlation (r^2^) in genotype frequencies between autosomal SNPs from six chromosomes. These were the same chromosomes that were used by Colonna et al. [18], i.e., chromosomes 1, 3, 7, 10, 18 and 22, using PLINK. For each population, average r^2^ between markers was calculated for bins of 5 kb length, running from 0–5 to 495–500 kb. An analogous index of the correlation coefficient for measuring LD measures disequilibrium on the basis of homozygosity [29]. While this measure be more accurate in the context of unphased data, we report standard r^2^ for consistency with existing literature.

### 2.6. IBD Sharing

Because higher SNP density increases power to confidently detect IBD segments, IBD sharing analysis was only applied to the two Dutch samples (TWN and NLD) that were both genotyped on the CytoSNP array. BEAGLE (version 3.0.4) [30] was used to determine haplotype phasing. Phased haplotypes were then analyzed with the GERMLINE program (version 1.5.1) [31] to identify segments of IBD. Because of the relatively low SNP density in our array, we require a minimum IBD segment length of 3 cm, allowing at most 1 mismatch in windows of 64 markers. The *p*-values were estimated empirically by permuting population labels and assessing significance on basis of the non-parametric Wilcoxon Rank Sum Test statistic (10,000 permutations). This method corrects for potential differences in sample size and is conservative with respect to outliers driving the signal. Additionally, we excluded regions with low SNP density (>20% overlap of sparse regions, >1 Mb per 50 SNPs).

### 2.7. Runs of Homozygosity (ROH)

Runs of homozygosity were identified using default PLINK homozyg settings (length = 1000 kb, #SNPs = 100, density = 50 kb/SNP, largest gap = 1000kb) in the combined dataset. ROHs were binned based on length: up to <1.49 Mb, 1.5–2.49 Mb, 2.5–4.99 Mb, 5–9.99 Mb and over 10 Mb in length. For each bin the proportion of individuals with one or more ROHs of that length was calculated. We performed a separate ROH analysis on the two Dutch samples (TWN and NLD) making use of the higher density of the array. Again, standard PLINK settings were used to find runs of homozygosity greater than 1Mb. The *p*-values of the differences in ROH between TWN and NLD were estimated using permutation based on Wilcoxon Rank-Sum Test statistics (both with 10,000 repetitions).

### 2.8. Effective Population Size

Recombination shortens IBD segments during meiosis, and the genetic length of shared haplotypes is probabilistically linked to the number of generations separating two individuals from their most recent common ancestor. In addition, when tracing the ancestry of a pair of individuals back in time, the chance of randomly finding their common ancestor is inversely proportional to the effective size of the analyzed population, with a smaller effective population size resulting on average on earlier common ancestors. Combining these to principles, one can use the length of IBD segments detected in sample to estimate the effective population size within and across populations at different time scales [32].

The following equation can be used to infer recent population size from IBD segments,

(1) $$N_{e}=\frac{50(1-f+\sqrt{1-f})}{uf}$$

where *N_e_* is the effective population size, f is the shared fraction of the genome and u is the minimum length of IBD segments included in the computation [32]. This estimator assumes constant population size.

Confidence intervals were estimated by bootstrapping the GERMLINE output [30,33] and *p*-values were estimated using permutation (both with 10,000 repetitions). To reflect recent population size we use segments >7 cm, corresponding roughly to a period of ~500 years (or 20 generations) ago as estimated in The Netherlands [33].

## 3. Results

**Figure 1:**
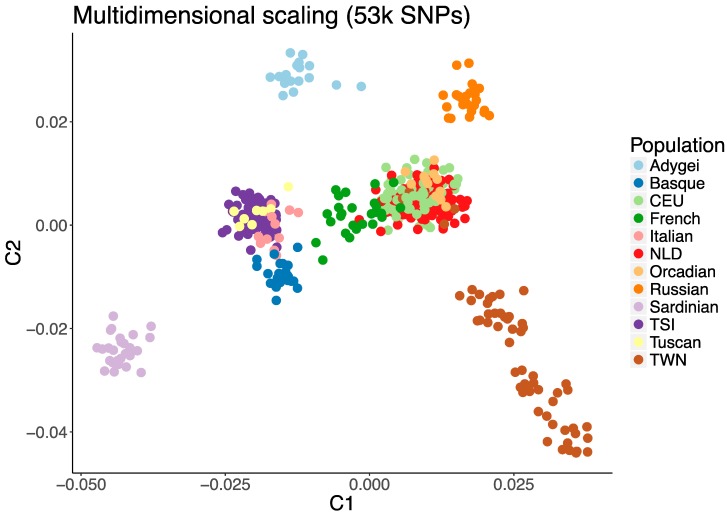
First two components (C1 and C2) of the multidimensional scaling (MDS) analysis. The TWN sample (brown) is clearly separated from the Dutch reference population (NLD, red). As expected, NLD colocalizes with CEU (light green). Further, Sardinia as well as the Adygei and Russian populations are separated from the other European reference samples and the CEU HapMap samples. SNPs; Single Nucleotide Polymorphims.

The measure of population stratification (by MDS analysis) clearly distinguished TWN from the other populations by the first two principal components (Figure 1). This was true for all but five subjects from TWN, who were indistinguishable from the Dutch controls (NLD). The results also recapitulated the known differences between the respective reference populations according to their geographic origin [24,34,35].

We calculated the genetic distances (F_ST_) between TWN and the different reference populations. The results (F_ST_ values and the 95% confidence interval(CI)) are shown in Table 1. All pair-wise comparisons between all populations are significant at a *p*-value threshold of 0.01, which is the lowest *p*-value obtainable by 100 permutations. Consistent to genealogical records, results based on genetic data show that the TWN population is closely related to the other European populations and genetically closest to the Dutch population (NLD).

**Figure 2:**
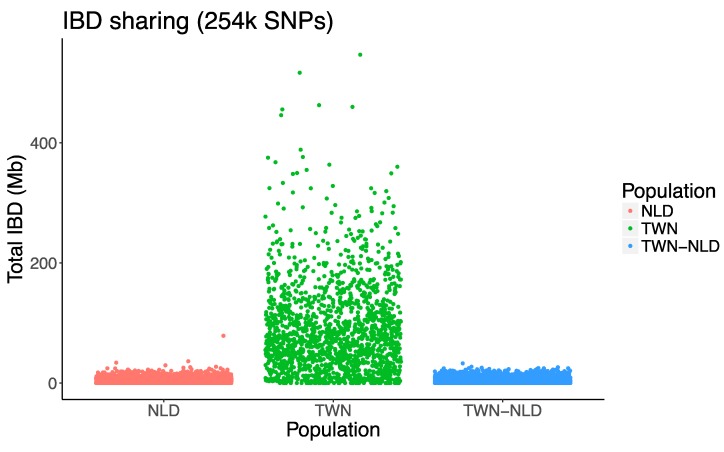
Average identical-by-descent (IBD) sharing within the control population (left), within the town (middle) and across the town and controls (right). Total IBD is increased within the Dutch town compared to the control population by a factor of 20 (with a mean of 4.34 Mb/pair IBD segments in the NLD sample compared to 87.25 Mb/pair in the TWN sample *p* < 0.0001).

We compared haplotype sharing in TWN and NLD and observed a significantly higher amount of IBD in TWN (Table 2; Figure 2). Our results show a mean of 4.34 Mb/pair IBD segments in the NLD sample compared to 87.25 Mb/pair in the TWN sample, (*p* < 0.0001). The increased haplotype sharing is driven both by the number of IBD segments (*p* < 0.0001), as well as by the average length of IBD segments (*p* < 0.0001) (Table 2). There is no significant increased IBD sharing across the two populations (*p* = 0.06) (Table 3) Figure B1 displays distributions of the average fraction of genome shared by IBD segments of a certain size per population. Due to the stringency of our GERMLINE settings, very few of these segments are likely to be false-positives. However, if we exclude regions with sparse genotyping, we get the same qualitative results, as can been seen in Table 4.

**Figure 3:**
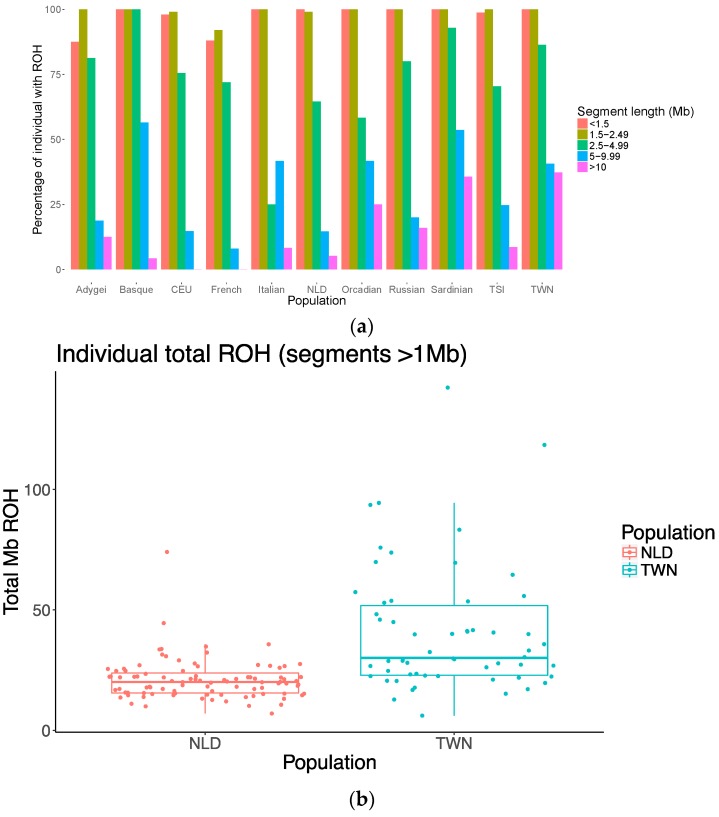
Runs of Homozygosity (ROH) (**a**) Proportion of individuals with one or more ROHs of a given length for the TWN population, NLD, and other European populations (HapMap and HDGP). ROHs were binned according to length: of up to 0.5–1.49, 1.5–2.49, 2.5–4.99, and 5–9.99 Mb in length, or over 10 Mb in length, plotted for each population. (**b**) Box plot depicting total length of ROH segments >1 Mb in the Dutch town versus Dutch controls (upper and lower hinges correspond to the 25th and 75th percentiles, and whiskers to 95% intervals). Each point indicates total ROH length (Mb) per individual. Samples from TWN have an increased average total ROH length (41 Mb ROH in TWN versus 21 in NLD, *p* < 0.0001).

The highest percentage of subjects with at least one very long ROH (>10 Mb) is found in the TWN sample (37%), followed by the Sardinians with 36% and the Orcadians with 25% (Figure 3a). The proportion of subjects with long ROHs decreases quickly to 16% in Russians, 13% in Adygei and below 10% for the remaining populations. The distribution of smaller ROHs is similar but not identical. For example, the highest percentage of ROH in the 5–9.99 Mb range is observed the Sardinians (54%) and the Basques (57%). In all populations, more than 50% of individuals have at least one 2.5–4.9 Mb ROH bin. Comparing TWN and NLD samples directly, we observe a significantly increased total length of long (>1 Mb) ROHs in individuals from TWN compared to NLD: an average total of 41 Mb ROHs in TWN versus 21 in NLD (*p* < 0.0001). We observe both an increased number of ROH segments, as well as the average length of segments (*p* < 0.0001 in both cases, see Table 5 and Figure 3b). We also observe a strong correlation between total length of ROH and the first two MDS components (C1 rho = 0.70, *p* < 2.2 × 10^–16^; and C2 rho = −0.71, *p* < 2.2 × 10^−16^, Spearman Rank correlation). That is, samples that are further away from the founding NLD population on the MDS plot (Figure 1), tend to have the greater total ROH length.

**Figure 4:**
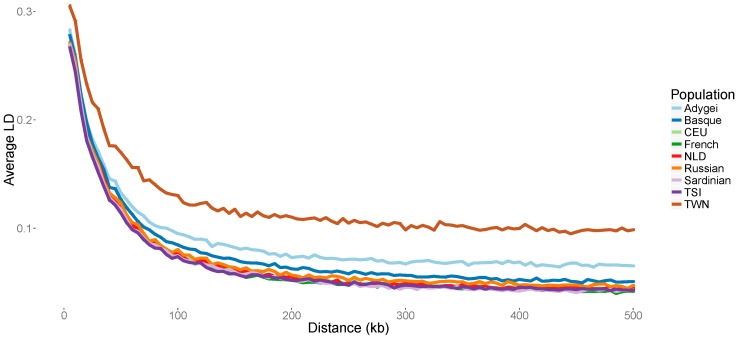
Decay of Linkage Disequilibrium (LD) over distance in kb. A random set of 25 individuals was drawn from the TWN, NLD, CEU and TSI populations to obtain equal sized sample sets. Populations with a sample size smaller than 20 were not analyzed (i.e., Adygei, Italy-Bergamo, Orcadian and Tuscan samples). *R^2^* was estimated for all SNP pairs within chromosomes 1, 3, 7, 10, 18 and 22. Results were averaged over distance using bins of 5 kb length and plotted against the upper limit of the bin. The first bin was of smaller size (1–5 kb).

We measured the decay of LD by genetic distance. The TWN population showed the highest levels of LD and the lowest rates of decay relative to genetic distance compared to all reference populations included in our study (Figure 4). Consistent with the above-mentioned IBD results, this effect is particularly visible over long recombination distances.

We observed a significant difference in recent effective population corresponding roughly to a period of ~500 years ago (assuming a generation length of 25 years), of the NLD and TWN populations (*p* < 0.0001). The recent effective population size of TWN is estimated to be 754 (with CI 95% (745.0, 763.7)) while the NLD sample has an effective population size of 148,086 (with CI 95% (139,840, 154,656)).

## 4. Discussion

We observed consistent and significant evidence that the TWN population is a genetic isolate within The Netherlands. This population could be readily distinguished from other European reference populations including a Dutch control sample using a multi-dimensional scaling analysis of genome-wide genotype data.

The measure of genetic distance (F_ST_) between the populations confirmed that TWN is a separate population but closest related to the Dutch. The actual F_ST_ values, despite being significantly different, are relatively low. This suggests that TWN became isolated from NLD only recently [27]. All genetic findings are in line with our historical knowledge of a founding population around 1000 A.D. in a geographically isolated region, a bottleneck event in the 17th century, and a subsequent expansion of its population. The extensive sharing of haplotypes IBD is remarkably high compared to other populations including other genetic isolates. A detailed comparison between TWN and its closest related (and founding) population, NLD, shows a 20-fold increase of IBD sharing.

A related measure, ROH distribution in TWN and NLD, shows a significant increase in number of homozygous regions as well and the length of these segments as well. Our results also indicate that individuals from TWN that are further removed from the founding population (NLD) on the MDS plot, show increased levels of homozygosity.

Comparing the TWN population to the reference populations in our study, including the known isolate Sardinia, we observe that TWN yielded the highest percentage of individuals with large (>10 Mb) regions of homozygosity and showed even lower levels of decay in LD by genetic distance. Slow decay of LD relative to genetic distance is used as a major indicator of genetic isolation in comparison to outbred populations [2] and has been shown to be present in confirmed genetic isolates such as villages from Sardinia [36] and the North-Italian Val Borbera valley [18]. The slow LD decay observed in our sample that was recruited from one single town is comparable with the findings in these Italian villages. The next highest proportion of ROH but without evidence of decreased levels of LD decay (at a population level) is found in Sardinia and the Basques. Interestingly, the increase of ROHs is in the latter population is most visible in the smaller segments between 5 and 10 Mb. This may be reflective of a different population history and effective population size compared to the TWN isolate. Although the genetic isolation of the Sardinians has been repeatedly demonstrated [36,37,38], whether the French Basques are a genetic isolate is still under debate [39,40].

Given all the evidence of TWN being a population isolate we also made an effort to estimate its effective population size as well as the number of generations since the bottleneck. While estimate of effective population size in the control sample NLD is close to 150,000, a number in concordance with earlier estimates from the Dutch population [33], the estimated number for the TWN population is several orders of magnitude smaller, around 750. With regard to IBD sharing and effective population size it should be noted that fixed thresholds (segments of >7 cm as in Francioli et al, 2014) were used instead of estimating recent demographic history using tools such as DoRIS [28,32,41]. The application of this method resulted in poor fits of the models (data not shown). One possible cause for this is that IBD segment lengths in the Dutch town are not exponentially distributed (see Figure B1), which is an underlying assumption of the method. This prevents us from accurately estimating population historical events and also affects the actual estimate of the effective population size of 754. While the estimate of *N_e_* = 754 and 500 years are thus not expected to be very accurate, the extent of the difference in effective population size between the TWN and NLD samples is striking.

Due to relatively low SNP density in the CytoSNP array we were not able to identify IBD segments below 3 cm with high confidence. It was therefore not possible to infer ancestral population size using segments 1–2 cm [33]. While all evidence (e.g., historical evidence, F_ST_ values and IBD sharing) points to recent isolation, it may be interesting to investigate potential differences in ancestral population size in future work.

Even within a small country as The Netherlands, differences in genetic variation can be mapped to geographical location [33,42]. The Dutch control samples used for this study were mostly collected from the larger cities in The Netherlands. It would be of interest for future genetic analyses to compare TWN with subjects from regions immediately surrounding the former island. Another interesting avenue for further study is a comparison between paternal and maternal history of the TWN population using genetic variation from the sex chromosomes and mitochondrial DNA.

In conclusion, we describe a new population isolate in The Netherlands showing extensive features of genetic isolation. The historical facts of relatively recent founding, geographic isolation for centuries, religious endogamy, bottleneck events resulting in limited number of founders and rapid population expansion, are all corroborated by the genetic evidence. The genetic history of this population, with currently one of the highest fertility rates in The Netherlands, makes it very valuable for genetic studies of human traits. This population is likely to have improved power for association studies for gene discoveries and will aid the identification of rare variants contributing to complex diseases.

## Figures and Tables

**Table 1:** F_ST_ values and 95% confidence interval (CI) between populations: Adygei ^1^, Basque ^1^, Utah residents with Northern and Western European ancestry from the CEPH collection ^2^ (CEU), Dutch controls (NLD), French ^1^, Russian ^1^, Sardinian ^1^ (Sard), Tuscans in Italy ^2^ (TSI) and Dutch town (TWN) samples.

	Adygei	Basque	CEU	NLD	French	Russian	Sard	TSI	TWN
Adygei	-	0.0182 (0.0179–0.0185)	0.0101 (0.0099–0.0103)	0.0108 (0.0105–0.0110)	0.0095 (0.0092–0.0098)	0.0116 (0.0112–0.0119)	0.0191 (0.0188–0.0194)	0.0078 (0.0075–0.0080)	0.01557 (0.0153–0.0159)
Basque	-	-	0.0081 (0.0079–0.0083)	0.0087 (0.0086–0.0089)	0.0068 (0.0066–0.0070)	0.0142 (0.0140–0.0146)	0.0129 (0.0127–0.0132)	0.0081 (0.0080–0.0083)	0.0135 (0.0133–0.0137)
CEU	-	-	-	0.0002 (0.0002–0.0002)	0.0004 (0.0003–0.0005)	0.0043 (0.0042–0.0044)	0.0119 (0.0117–0.0121)	0.0035 (0.0034–0.0036)	0.0044 (0.0043–0.0045)
NLD	-	-	-	-	0.0008 (0.0007–0.0010)	0.0047 (0.0046–0.0049)	0.0126 (0.0124–0.0128)	0.0040 (0.0039–0.0041)	0.0040 (0.0039–0.0041)
French	-	-	-	-	-	0.0053 (0.0051–0.0055)	0.0091 (0.0089–0.0093)	0.0018 (0.0017–0.0020)	0.0053 (0.0052–0.0055)
Russian	-	-	-	-	-	-	0.0197 (0.0194–0.0200)	0.0090 (0.0089–0.0092)	0.0089 (0.0088–0.0091)
Sard	-	-	-	-	-	-	-	0.0067 (0.0065–0.0069)	0.0175 (0.0173–0.0178)
TSI	-	-	-	-	-	-	-	-	0.0086 (0.0084–0.0087)

^1^ = HGDP dataset; ^2^ = HapMap III dataset.

**Table 2:** IBD sharing within NLD and TWN samples.

	# Samples	# Pairs	# IBD Segments	Mean/Median/SD # IBD Segments/Pair	Mean/Median/SD Size (Mb)/Segment	Mean/Median/SD Size (Mb)/Pair
NLD	92	4186	4548	1.09/1/1.05	3.99/3.57/1.36	4.34/3.5/4.57
TWN	58	1653	15444	9.34/8/6.45	9.34/7.22/8.20	87.25/71.99/73.88
*p*-value				<0.0001	<0.0001	<0.0001

SD, standard deviation.

**Table 3:** IBD sharing between CTRL and TWN pairs.

	# Samples	# Pairs	# IBD Segments	Mean/Median/SD # IBD Segments/Pair	Mean/Median/SD Size (Mb)/Segment	Mean/Median/SD Size (Mb)/Pair
NLD-TWN	92	2622	3822	1.45/1/1.12	3.92/3.57/1.09	5.68/3.64/4.67

**Table 4:** IBD sharing within NLD and TWN samples (excluding sparsely genotyped regions).

	# Samples	# Pairs	# IBD Segments	Mean/Median/SD # Segments/Pair	Mean/Median/SD Size (Mb)/Segment	Mean/Median/SD Size (MB)/Pair
NLD	92	4186	2648	0.63/0/0.80	3.93/3.57/1.36	2.48/0/3.41
TWN	58	1653	9802	6.05/5/4.43	10.01/7.22/8.20	59.37/46.85/55.63
*p*-value				<0.0001	<0.0001	<0.0001

**Table 5:** Long (>1 Mb) ROH in individuals from TWN compared to NLD.

	# Samples	Mean/Median/SD # ROH Segments/Pair	Mean/Median/SD Size (Mb)/Segment	Mean/Median/SD ROH Total Length (Mb)
NLD	92	11.92/11/2.97	1.73/1.64/0.38	20.99/20.04/8.55
TWN	58	15.17/14/4.80	2.64/2.13/1.34	40.94/30.09/26.51
*p*-value		<0.0001	<0.0001	<0.0001

**Figure A1:**
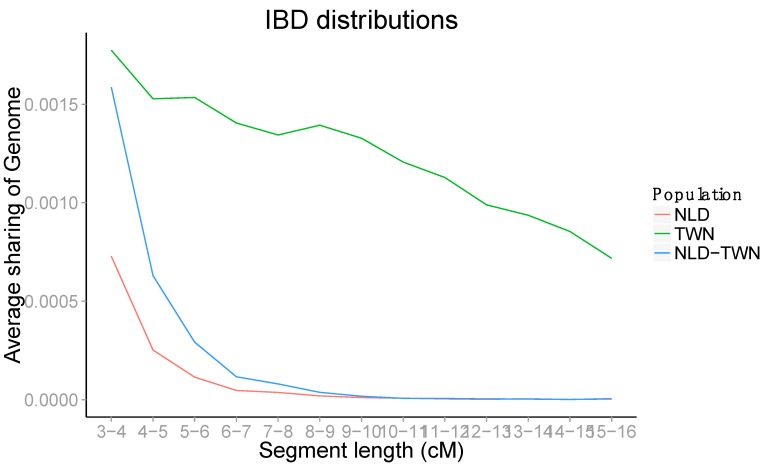
Distributions of the average fraction of genome shared (y-axis) by segments of a certain size (x-axis) per population. While NLD and TWN-NLD appear to follow an exponential distribution, the TWN population shows excessive sharing across segment lengths.

## Appendix A. Quality Control Pipeline

Because samples randomly drawn from an isolated population have a higher chance of being somehow related, a strict quality control (QC) pipeline was adopted. First, we performed separate QC on all datasets (Part 1). Next, we merged the data and performed subsequent QC in two parts. In Part 2a all datasets were included that were later used for F_ST_, MDS, ROH and LD-decay. In Part 2b, only TWN and NLD were merged for analyses involving a higher SNP density (ROH comparison between TWN and NLD, IBD sharing, effective population size estimation). The QC pipelines are detailed below.

### Appendix A.1. Part 1

We applied strict measures of quality control (QC) for all datasets separately using PLINK 1.07 [26]. The following thresholds were used: missingness at <0.05%, genotype call rate of >99%, minor allele frequency (MAF) of <0.1 and a Hardy-Weinberg equilibrium (HWE) *p*-value >0.00001. Subsequent pruning of SNPs was performed at a pair-wise genotypic r^2^ < 0.2 within sliding windows of 50 SNPs (with a 5-SNP increment between windows using the default settings in PLINK). Next, the dataset was screened for inconsistencies at the subject level: those with a gender mismatch between phenotype and genotype (2 NLD) using PLINK, those with high heterozygosity (F > ±3 SD: 1 NLD, 2 CEU, 1 TSI, 1 French, 1 Basque, 1 Adygei), closely related individuals (pairwise proportion identity-by-decent (pi-hat) >0.1: 13 TWN; 1 NLD; 2 Orcadian) or outliers (>2 or 3 SD, depending on distribution, on the first two principal components (PC) of multidimensional scaling (MDS) analysis: 4 NLD, 6 CEU, 4 TSI, 1 French). Based on these criteria, we removed a number of subjects from the original datasets (13 TWN, 8 NLD, 8 CEU, 5 TSI, 2 French, 1 Basque, 1 Adygei, 2 Orcadian), so that 59 TWN, 96 NLD, 102 EU, 81 TSI, 25 French, 23 Basque, 28 Sardinian, 25 Russian, 16 Adygei, 12 Italy-Bergamo and 12 Orcadian samples remained. On the remaining samples the following filters were applied before merging: genotype call rate of 99%, missingness >0.02, MAF <0.01, HWE >0.000001. The remaining numbers of SNPs were 255,314, 255,889, 996,761 and 992,887 for the TWN, NLD, CEU and TSI populations, respectively, and between 559,084 and 586,157 for the HGDP populations.

### Appendix A.2. Part 2a

For the analyses involving all datasets (F_ST_, MDS, ROH and LD-decay analyses) subsequent QC was performed after merging the data: removal of samples >0.1% missingness, genotype call rate >90%, MAF >0.01 and HWE *p*-value >0.00005. These steps resulted in a set of 112,444 overlapping SNPs that were used for analysis. For the MDS analysis SNPs were pruned with a pairwise genotypic r^2^ > 0.2 within sliding windows of 50 SNPs (with a 5-SNP increment between windows) was applied, resulting in a set of 53,490 high-confidence approximately independent SNPs. For F_ST_ analysis we only included populations from which the sample size is greater than 15 (e.g., Italy-Bergamo and Orcadian were excluded).

### Appendix A.3. Part 2b

For analyses involving a higher SNP density (ROH comparison between TWN and NLD, IBD sharing, effective population size estimation) we only included TWN and NLD samples genotyped on the same array. Similar QC was performed after merging the two datasets: MAF 0.01, genotype call-rate 0.01, missingness 0.02 and HWE *p*-value >0.000001. A total of 254,582 SNPS in 58 individuals from TWN and 92 from the NLD sample were included in the subsequent.
